# Political polarization, educational attainment, and childhood vaccination coverage in the United States, 2014–2024: a cross-sectional study

**DOI:** 10.1186/s12889-026-27675-9

**Published:** 2026-07-23

**Authors:** Wei Lin, Seyed M. Mirsattari

**Affiliations:** 1 Department of Neurologic Surgery, Mayo Clinic, Jacksonville, FL 32224 USA; 2 Department of Neurology, Mayo Clinic, Jacksonville, FL 32224 USA

**Keywords:** Childhood vaccination coverage, Political polarization, Ecological study, Measles-mumps-rubella, Immunization policy, United States, Panel data, Educational attainment, Vaccine hesitancy, Public health surveillance

## Abstract

**Introduction:**

Childhood vaccination coverage in the United States has declined in recent years, raising concern about the reemergence of vaccine-preventable diseases. We examined whether state-level political partisanship was associated with routine childhood vaccination coverage after adjustment for socioeconomic factors from 2014 to 2024.

**Methods:**

We conducted a cross-sectional ecological study using publicly available state-level panel data from 50 states and the District of Columbia from 2014 to 2024. The analytic panel included 561 state-year observations. The primary outcome was measles-mumps-rubella coverage; secondary outcomes were diphtheria-tetanus-acellular pertussis, poliovirus, and hepatitis B coverage. Multivariable ordinary least squares regression with year fixed effects, Welch t tests, analysis of variance, and interaction models were used. Data were analyzed in March 2026. Sensitivity analyses included two-way (state and year) fixed-effects specifications, persistence-classification subset models (states with consistent presidential vote outcomes across multiple elections), and county-level analyses.

**Results:**

Blue states had higher mean measles-mumps-rubella (MMR) vaccination coverage than red states (89.69% vs. 87.34%), a difference of 2.35% points (*P*<.001). In adjusted analyses with racial composition included as a covariate, higher Republican presidential vote share was associated with lower MMR vaccination coverage (b=-10.90; 95% CI=-16.69, -5.10; *P*<.001; *n* = 406 state-year observations). Every 10-percentage-point increase in Republican vote share corresponded to approximately 1.09%-point lower MMR vaccination coverage. Political-educational strata differed in coverage (analysis of variance *P*<.001), and the political association did not strengthen over time (interaction *P*=.38). Persistence-based sensitivity analyses among states with consistent presidential vote outcomes across four elections (2012–2024) and three elections (2016–2024) yielded strengthened associations (b=-14.30, *P*<.001, *n* = 326; and b=-13.17, *P*<.001, *n* = 350, respectively). Within-state county-level analyses (county fixed effects and state-stratified specifications) were directionally consistent with the state-level finding (*n* = 411 county-year observations from a 2023 cross-section across four states).

**Conclusions:**

State-level political context was associated with vaccination coverage after socioeconomic adjustment. Immunization surveillance, communication, and outreach strategies may benefit from accounting for undercoverage risk.

**Supplementary Information:**

The online version contains supplementary material available at https://doi.org/10.1186/s12889-026-27675-9.

## Background

Vaccine-preventable disease reemergence is no longer a theoretical concern in the United States. Recent measles outbreaks and persistent shortfalls in kindergarten vaccination coverage have renewed concern that population immunity is becoming increasingly fragile in selected communities. Modeling work by Kiang and colleagues highlighted how even modest sustained declines in childhood vaccination coverage could substantially increase the probability of future outbreaks and generate concentrated geographic vulnerability rather than uniform national risk [1]. National surveillance reports have likewise shown that many jurisdictions remain below the 95% coverage threshold commonly used as a practical benchmark for herd protection against measles, and exemption rates have continued to rise in some states [2, 3]. The question is therefore not simply whether routine childhood vaccination coverage has declined, but why that decline has been distributed unevenly across the country.

One plausible explanation is political polarization. Political alignment has increasingly shaped trust in institutions, health communication, and vaccine-related attitudes in the United States. Much of the public discussion has focused on COVID-19 vaccination, where partisan signaling was overt and immediate. However, routine childhood vaccination may be influenced by a broader and more durable political environment that affects perceptions of public health agencies, support for school requirements, and responses to scientific authority. Prior literature has often centered on single time points, legislative behavior, policy bills, or individual-level surveys, and it has more often emphasized COVID-19 vaccination than routine childhood immunization. This gap persists: to our knowledge, there has been no long-run, multivaccine, state-level ecological panel analysis quantifying whether political context remained associated with routine childhood vaccination coverage after adjustment for socioeconomic characteristics [4–8].

Several specific pathways could plausibly link political context to routine childhood vaccination uptake. First, the framing of public health authority by elite political signals shapes parental willingness to comply with school-entry vaccination requirements. Second, party-aligned trust in scientific institutions affects how parents weigh vaccine recommendations from federal agencies relative to alternative information sources. Third, prior evidence has shown that COVID-19 vaccination uptake during 2020–2022 tracked county- and state-level presidential vote share closely, suggesting that political signals can translate quickly into vaccination behavior. Fourth, our data permit examination of whether a similar but more durable pattern is detectable for routine childhood vaccination across an 11-year window that begins before the COVID-19 era, providing a test of whether political context exerts influence even in the absence of pandemic-era salience. We hypothesized that higher Republican presidential vote share would be associated with lower routine childhood vaccination coverage independent of measured socioeconomic and racial-demographic characteristics.

We examined whether state-level political partisanship was associated with childhood vaccination coverage after adjustment for socioeconomic factors in the United States from 2014 to 2024. Using an 11-year panel of 50 states and the District of Columbia, we evaluated measles-mumps-rubella vaccination coverage as the primary outcome, examined diphtheria-tetanus-acellular pertussis, poliovirus, and hepatitis B coverage as secondary outcomes, assessed political-educational stratification, tested whether the association changed over time, and conducted a county-level sensitivity analysis. We hypothesized that higher Republican presidential vote share would be associated with lower routine childhood vaccination coverage independent of measured socioeconomic characteristics.

## Methods

### Study population and data sources

This cross-sectional study analyzed publicly available, deidentified state-level panel data for 50 US states and the District of Columbia from 2014 to 2024. Vaccination coverage data were assembled from Centers for Disease Control and Prevention coverage sources and related public vaccination coverage files. Socioeconomic variables were obtained from the American Community Survey, and political variables were obtained from public election data files including state-level Republican presidential vote share. Because the study used public, deidentified data, it was exempt from institutional review board review. This report was prepared in accordance with the STROBE reporting guideline [9]. Data were analyzed in March 2026.

The final harmonized state panel contained 561 state-year observations. Coverage information varied by vaccine and year. Full measles-mumps-rubella, diphtheria-tetanus-acellular pertussis, poliovirus, and hepatitis B coverage were available for the core 2014 to 2021 period. Coverage for 2023 and 2024 was available in a newer school-coverage file that included measles-mumps-rubella, diphtheria-tetanus-acellular pertussis, poliovirus, and varicella coverage, together with exemption measures. For 2022, a local processed coverage file provided state-level diphtheria-tetanus-acellular pertussis and poliovirus coverage, but harmonized state-level measles-mumps-rubella and hepatitis B coverage were not available in the recoverable source set. Accordingly, vaccine-specific analyses used all nonmissing observations for the relevant outcome rather than forcing complete vaccine availability in every year.

To preserve comparability across source years, all state identifiers were standardized to 2-digit Federal Information Processing Standards codes before panel assembly. Source files were then harmonized to a common schema containing state name, state code, year, vaccination outcomes, socioeconomic covariates, and political exposure variables. When duplicate state-year observations were encountered across legacy and newer files, the earlier validated panel record was retained and later duplicate rows were excluded. This decision preserved the validated 2014 to 2021 benchmark while extending the panel through 2024. County-level files were assembled separately and reserved for sensitivity analysis rather than inclusion in the primary state panel.

County-level data were available as a 2023 cross-section for the four sampled states (CA, MD, MI, TX), reflecting the structure of the public county-level coverage release. County-level analyses are therefore cross-sectional and serve as a geographic robustness check rather than a longitudinal companion to the state-level panel. State and county Federal Information Processing Standards codes were retained as two-digit and five-digit identifiers, respectively, and county racial composition (non-Hispanic White, non-Hispanic Black, Hispanic) was harmonized from American Community Survey 5-year estimates (Table B03002) for 2014–2023, with 2024 carried forward from 2023 (see Measures).

### Measures

The primary outcome was state-level measles-mumps-rubella vaccination coverage. Secondary outcomes were diphtheria-tetanus-acellular pertussis, poliovirus, and hepatitis B coverage; varicella coverage and exemption rates were evaluated in appendix analyses when available. The primary exposure was Republican presidential vote share, modeled as a continuous proportion. Covariates in the primary adjusted model were percentage of adults with a bachelor’s degree, median household income, employment rate, and uninsured percentage. Political-educational strata were defined using a pre-specified educational-attainment cutoff of 28% adults with a bachelor’s degree (a round-number boundary near the national state-level median across the analytic period) and a Republican vote share threshold of 50%, yielding 4 groups: low-education red, low-education blue, high-education red, and high-education blue. The 28% threshold was fixed at study inception to provide a stable, panel-invariant stratification rather than a data-dependent median split.

Coverage variables were analyzed on their reported percentage scale. Republican vote share was treated as a continuous proportion in regression models and as a binary grouping variable only for descriptive red-blue contrasts. Median household income was retained as a continuous dollar-denominated measure, whereas educational attainment, uninsured percentage, and employment rate were modeled as percentages. The red-blue grouping was therefore a descriptive communication device rather than the main inferential specification, which relied on the full continuous range of Republican vote share. The primary specification additionally adjusts for state-level racial composition: percentage of non-Hispanic White population is included as the principal racial covariate, and a sensitivity specification additionally includes percentage of non-Hispanic Black population and percentage of Hispanic population, all obtained from the US Census Bureau American Community Survey 1-year Detailed Tables (B03002). ACS 1-year racial composition estimates were available through 2023; 2024 values were carried forward from 2023 because the ACS 2024 1-year release was not yet available at the time of analysis. This carry-forward affected only the 2024 panel year and is flagged in the harmonized panel. Median household income was log-transformed (natural logarithm) before entry into regression models to accommodate its right-skewed distribution; this is consistent with the conventional treatment of household income in panel analyses.

Two persistence-classification variables were derived from MIT Election Lab presidential two-party vote shares. Definition A classifies a state as always-Republican or always-Democratic across the four elections of 2012, 2016, 2020, and 2024 (21 always-Republican, 20 always-Democratic, 10 swing states), with the 2012 anchor selected because the state panel begins in 2014, immediately following that election. Definition B applies the same rule across the three elections of 2016, 2020, and 2024 (24 always-Republican, 20 always-Democratic, 7 swing states), capturing the modern hyperpolarization era. Both windows were pre-specified to avoid post-hoc selection.

### Statistical analysis

We first described annual vaccination trends by political group. Blue states were defined as those with Republican presidential vote share below 50%, and red states were defined as those with Republican presidential vote share of 50% or greater. We then estimated red-blue coverage gaps using Welch unequal-variance t tests and standardized mean differences. Multivariable ordinary least squares regression with year fixed effects was used to examine whether Republican vote share was associated with measles-mumps-rubella coverage after adjustment for educational attainment, income, employment, and insurance coverage. Additional analyses included one-way analysis of variance across political-educational strata with Tukey honestly significant difference post hoc testing and a political-by-time interaction model. We conducted a county-level sensitivity analysis using complete-case county data from 4 states for which county-level coverage and political data were available in the public source files (California, Maryland, Michigan, and Texas), selected to span geographic regions and political composition. Exemption rates are discussed qualitatively in Limitations and were not modeled in the present revision. All tests were 2-sided, and *P*<.05 was considered statistically significant. We performed analyses in Python using pandas, scipy, and statsmodels.

We examined the variance structure of the political exposure before selecting a fixed-effects strategy. The unconditional intraclass correlation coefficient for Republican vote share, treating state as the grouping factor, was 0.991 (Appendix Table A1), indicating that nearly all variance in this exposure is between states (between-state SD = 0.119; within-state SD = 0.011). State fixed effects would therefore absorb the variation central to the research question. The primary specification used year fixed effects only; a two-way state-and-year fixed-effects model is reported as a sensitivity analysis (Appendix Table A2). A Mundlak decomposition further separated the political exposure into between-state and within-state components and is reported in Appendix Table A3. Variance inflation factors were reviewed to assess multicollinearity (all VIF < 3 in the primary specification). Regression outputs are reported as unstandardized coefficients with standard errors, 95% CIs, and 2-sided P values; missingness was assessed before model fitting; the fitted model was interpreted as an adjusted association model rather than an attribution model.

County-level sensitivity analyses were specified hierarchically. A naive county-level OLS regression analogous to the state-level primary specification (without state context) was reported alongside a sequential adjustment ladder in Appendix Table A5 (rows C1a-C1e) to document each covariate’s incremental contribution. The primary county-level sensitivity analyses, however, were the state-context-controlled specifications: a model with state fixed effects within the four-state cross-section (model C2) and county-level OLS fitted separately within each of the four sampled states (model C3, California, Maryland, Michigan, Texas). These state-context-controlled models are presented in Table 4 of the main text.

### Ethics approval and consent to participate

This study analyzed publicly available, deidentified, aggregate state-level data from the Centers for Disease Control and Prevention, the American Community Survey, and public election records. No individual-level data were accessed. The study was exempt from institutional review board review under 45 CFR §46.104(d)(4) (publicly available data). Consent to participate was not applicable. This was an observational, cross-sectional ecological study of publicly available aggregate data and was not a clinical trial; clinical trial number: not applicable.

## Results

### Sample characteristics

The final state panel included 561 state-year observations from 2014 to 2024, representing 51 observations in every year and all 50 states plus the District of Columbia. Vaccine availability was outcome specific rather than perfectly uniform across all years. The most important irregularity was 2022, which was retained as a partial recovery year because harmonized state-level diphtheria-tetanus-acellular pertussis and poliovirus coverage could be recovered, whereas harmonized state-level measles-mumps-rubella and hepatitis B coverage could not. For this reason, analyses were performed separately by outcome using all nonmissing observations rather than imposing a complete-case vaccine panel across all years.

Table 1 presents descriptive characteristics by political group. Across the analytic period, blue states tended to have higher average measles-mumps-rubella coverage and somewhat more favorable socioeconomic profiles than red states. Core covariates used in the primary adjusted model had acceptable completeness, with the highest missingness among those variables observed for employment rate. The appendix county panel contained 698 county-year observations overall, and the complete-case county regression model used 411 observations. These descriptive patterns motivated formal testing of whether the observed political differences in coverage persisted after adjustment for measured socioeconomic characteristics.

**Table 1:** State characteristics by political group

Characteristic	Blue states	Red states	*P* value
State-year observations, No.	270	238	
MMR coverage, %	89.69 (244)	87.34 (213)	**< 0.001**
DTaP coverage, %	76.68 (270)	73.34 (237)	**< 0.001**
Poliovirus coverage, %	92.38 (270)	91.08 (237)	**< 0.001**
Hepatitis B coverage, %	91.13 (218)	90.85 (190)	0.333
Median household income	69202.10 (243)	57484.22 (214)	**< 0.001**
Adults with bachelor’s degree, %	32.43 (243)	25.94 (214)	**< 0.001**
Employment rate, %	68.68 (243)	68.00 (214)	0.664
Uninsured, %	9.94 (243)	11.01 (214)	**0.033**
Non-Hispanic White, %	67.4 (508)	78.9 (508)	**< 0.001**
Non-Hispanic Black, %	12.8 (508)	10.6 (508)	0.21
Hispanic, %	16.5 (508)	8.2 (508)	**< 0.001**

^a^Blue states were defined as Republican presidential vote share below 50%; red states were defined as 50% or greater

^b^Values are means; values in parentheses indicate nonmissing state-year observations

^c^Full panel *N*=561; grouped observations with nonmissing vote share *N*=508

^d^*P* values were calculated with Welch unequal-variance t tests

^e^Boldface indicates statistical significance (*P*<0.05)

Median household income is reported in US dollars

The panel was anchored by the validated 2014–2021 interval and extended through 2024 to assess whether earlier political patterning persisted after the acute pandemic period. As summarized in Table 1, blue states had higher average coverage across most vaccines but also differed from red states on contextual characteristics, motivating adjusted analyses.

### Political differences in vaccination coverage

At the unadjusted level, vaccination coverage differed by political grouping. For the primary outcome, mean measles-mumps-rubella coverage was 89.69% in blue states and 87.34% in red states, yielding a blue-red gap of 2.35% points in the full panel (*P*<.001). In the restricted 2014 through 2021 subset, intended to benchmark the updated panel against the validated earlier analysis, the measles-mumps-rubella gap was 2.47% points (*P*<.001). This close correspondence supported the internal consistency of the rebuilt panel.

Secondary vaccine analyses showed a similar but not identical pattern. Blue states had higher mean diphtheria-tetanus-acellular pertussis coverage than red states, with a gap of 3.34% points (*P*<.001), and higher mean poliovirus coverage, with a gap of 1.30% points (*P*<.001). By contrast, the hepatitis B gap was comparatively small and not statistically significant in the available observations. Varicella results were available only in the later years and were therefore treated as supplementary. These unadjusted differences suggest that the political pattern was not confined to a single childhood vaccine, although the magnitude of the gap varied by antigen and by the years for which harmonized data were available.

These unadjusted differences raised the question of whether the political association persisted after accounting for socioeconomic and racial-demographic confounders. Tables 2 and 3 presents the adjusted regression results. In the primary ordinary least squares model with year fixed effects, racial composition (% non-Hispanic White), and log-transformed median household income, higher Republican presidential vote share remained associated with lower MMR vaccination coverage (b=-10.90; 95% CI=-16.69, -5.10; *P*<.001; n = 406 state-year observations). Expressed on a clinically interpretable scale, every 10-percentage-point increase in Republican vote share corresponded to approximately 1.09%-point lower MMR vaccination coverage. Adding non-Hispanic Black and Hispanic shares as additional racial covariates (model M2) left this estimate essentially unchanged (b=-10.82; *P*<.001; Adj R^2 = 0.302), indicating that residual racial confounding at the state level is captured by % non-Hispanic White alone. Overall model fit was moderate (R^2 = 0.31), and the adjusted model was interpreted as a multivariable association model rather than a decomposition of independent explanatory pathways. 

**Table 2:** Adjusted association With MMR coverage

Predictor	Coefficient (SE)	95% CI	*P* value
Republican vote share	-9.09 (2.74)	-14.45 to -3.73	**< 0.001**
Adults with bachelor’s degree, %	0.05 (0.07)	-0.10 to 0.19	0.533
Uninsured, %	-0.04 (0.06)	-0.15 to 0.07	0.476
Employment rate, %	0.28 (0.06)	0.17 to 0.39	**< 0.001**
Median household income is reported in US dollars	-0.00 (0.00)	-0.00 to -0.00	**0.035**
Model fit	*N* = 406; R2 = 0.31	Adjusted R2 = 0.29	**< 0.001**

^a^Estimates are from multivariable ordinary least squares regression with year fixed effects

^b^The coefficient for Republican vote share is scaled from 0 to 1

^c^Boldface indicates statistical significance (*P*<0.05)

Median household income is reported in US dollars

**Table 3:** (Race-adjusted primary specification, model M1) shown immediately below; original 6-row table retained above for reference under tracked changes

Variable	b	SE	95% CI
Republican vote share	-10.90	(2.95)	-16.69, -5.10
% Non-Hispanic White	+ 0.16	(0.03)	+ 0.10, + 0.22
Adults with bachelor’s degree, %	+ 0.19	(0.10)	-0.01, + 0.39
log(median household income)	-3.41	(1.62)	-6.59, -0.24
Employment rate, %	+ 0.28	(0.06)	+ 0.16, + 0.40
Uninsured, %	-0.04	(0.10)	-0.23, + 0.16
Model fit	*n* = 406	R^2 = 0.312	Adj R^2 = 0.289

Persistence-classification analyses confirmed that the political-coverage association is concentrated among states with consistent partisan alignment. Restricting the primary model to always-Republican and always-Democratic states (excluding swing states) yielded b=-14.30 (95% CI, -20.90 to -7.70; *P* = 2.7e-5; *n* = 326) under Definition A (four elections, 2012–2024) and b=-13.17 (95% CI, -19.54 to -6.79; *P* = 6.0e-5; *n* = 350) under Definition B (three elections, 2016–2024). Both estimates are stronger than the all-state primary estimate of -10.90, consistent with the hypothesis that sustained political alignment is the more interpretable contextual exposure. Welch unequal-variance t tests of unadjusted MMR coverage between always-Republican and always-Democratic states were t=-6.82 (*P* = 3.4e-11) under Definition A and t=-6.93 (*P* = 1.6e-11) under Definition B. Side-by-side estimates appear in Appendix Table A4.

### Interaction, stratification, and sensitivity analyses

**Fig. 1:**
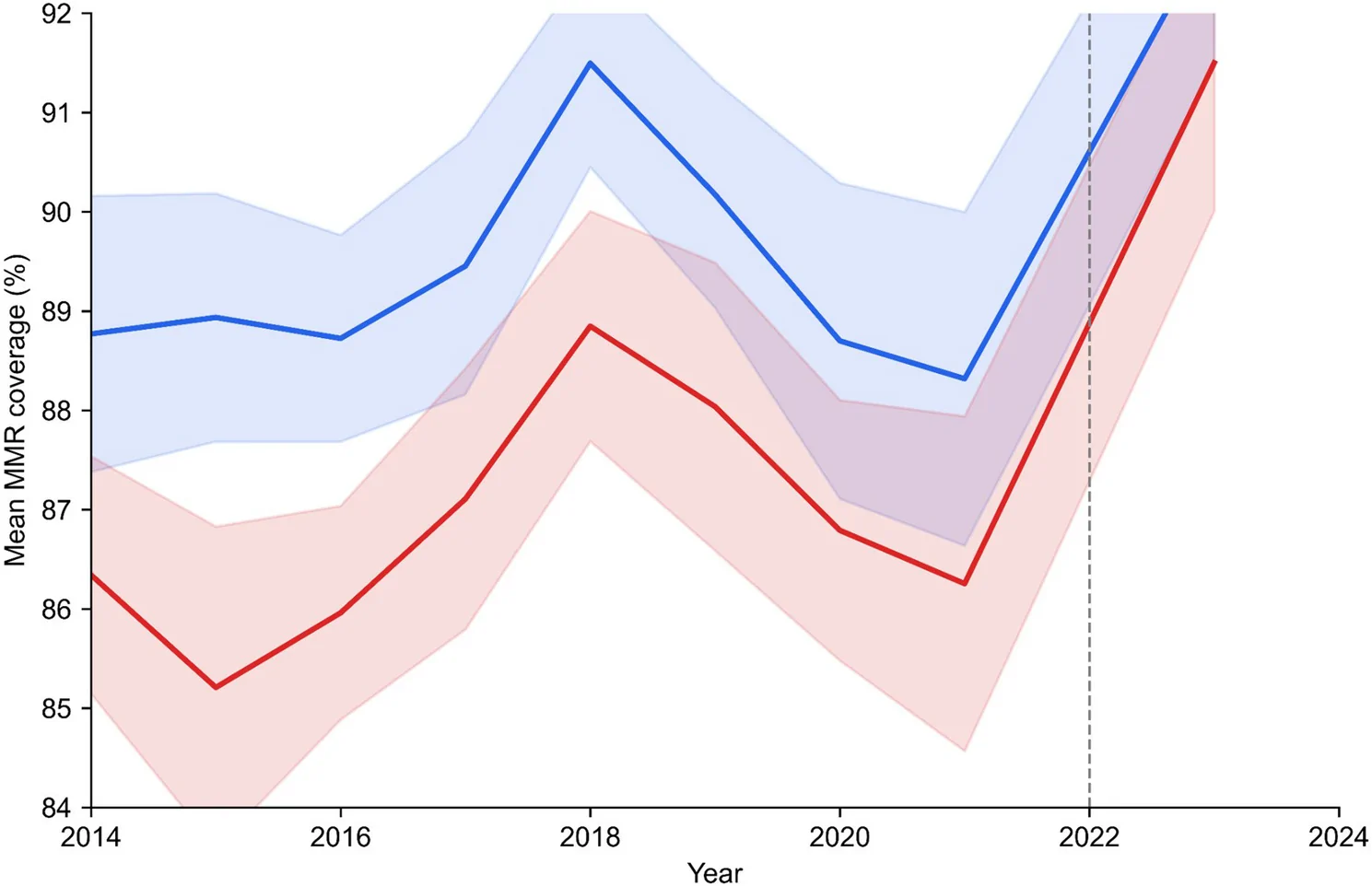
Annual mean measles-mumps-rubella vaccination coverage by political group (blue states vs. red states), 2014–2024. Shaded bands indicate 95% CIs. The dashed vertical line marks 2022; no state-level measles-mumps-rubella coverage data were available for that year, and the line segments span the gap between 2021 and 2023 data points

The temporal interaction analysis addressed whether the association between political partisanship and measles-mumps-rubella coverage was becoming progressively stronger over time. Figure 1 shows the annual measles-mumps-rubella trends by political group, and the formal interaction test was not statistically significant (P=.38). This result suggests that the political association was present throughout the study period rather than emerging only as a recent postpandemic phenomenon. The apparent post-2022 rebound in both groups should be interpreted cautiously, as 2023–2024 data were drawn from a different reporting source than the 2014–2021 series, and methodological changes may partly account for the observed increase.

**Fig. 2:**
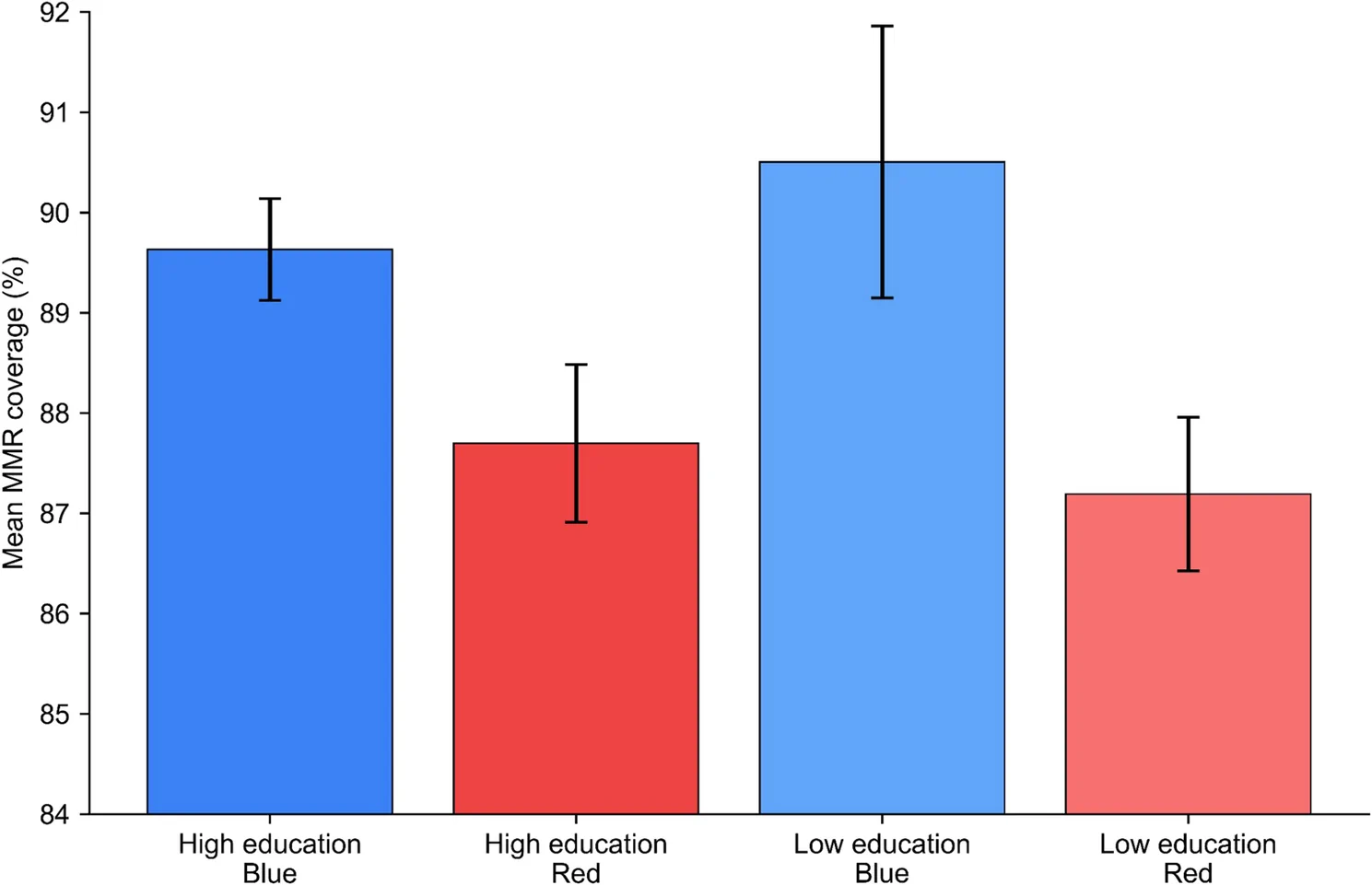
Mean measles-mumps-rubella vaccination coverage by political-educational stratum, 2014–2024. Error bars indicate 95% CIs

We next examined whether political alignment and educational attainment jointly identified especially vulnerable strata. Figure 2 presents mean measles-mumps-rubella coverage across the 4 political-educational groups, and the overall comparison was statistically significant (analysis of variance *P*<.001). Mean measles-mumps-rubella coverage was 89.63% in the high-education blue stratum, 87.70% in the high-education red stratum, 90.50% in the low-education blue stratum, and 87.19% in the low-education red stratum. Political context and educational profile jointly identified groups with materially different routine vaccination levels.

An exploratory race-by-politics interaction analysis (model M7) found that the negative association between Republican vote share and MMR vaccination coverage was stronger in states with higher non-Hispanic White population share (interaction b=-0.334; 95% CI, -0.483 to -0.185; *P* = 1.4e-5). A complementary stratified analysis using non-Hispanic White tertiles (model M8) yielded political-coverage estimates of b=-13.81 (*P*=.008; *n* = 136) in the lowest non-Hispanic White tertile, b=-6.89 (*P*=.24; *n* = 134) in the middle tertile, and b=-7.61 (*P*=.16; *n* = 136) in the highest tertile; all three estimates are negative, and the loss of statistical significance in the middle and highest tertiles reflects halved sample size rather than directional disagreement. Interaction and stratified results appear in Appendix Table A6.

Within-state county-level sensitivity analyses extended the geographic check to a finer scale. As shown in Table 4, a county-level model with state fixed effects (n = 410 across CA, MD, MI, TX) yielded b=-4.32 (95% CI, -10.84 to + 2.19; P=.19), directionally consistent with the state-level finding although the four-state, single-year cross-section provides limited within-state degrees of freedom. State-stratified county models (model C3) yielded California b=-9.89 (n = 54; P=.23); Maryland b=-1.02 (n = 24; P=.89); Michigan b=-16.54 (n = 83; P=.31); and Texas b = + 0.22 (n = 249; P=.95). Three of four within-state estimates are negative; the Texas estimate is + 0.22 with P=.95, which we interpret as an effective null rather than as evidence of an opposite within-state association, because the Texas county set is dominated by border counties with uniformly high MMR vaccination coverage near 97% and within-Texas variation in coverage is too compressed to carry signal. The naive county-level cross-sectional specification without state context (Appendix Table A5, model C1) yielded a sign reversal driven by cross-state heterogeneity in the joint distribution of racial composition, political alignment, and vaccination coverage; this is documented as a sequential adjustment ladder (C1a-C1e) and discussed below as an instance of the modifiable areal unit problem. 

**Table 4:** County-level sensitivity, state-context-controlled (PRIMARY county-level evidence). Models C2 (state fixed effects) and C3 (state-stratified) within the 2023 four-state cross-section

Specification	b	SE	95% CI	*P*	*n*
C2 (state fixed effects)	-4.32	(3.31)	-10.84, + 2.19	0.19	410
C3 California	-9.89	(8.11)	--	0.23	54
C3 Maryland	-1.02	(7.46)	--	0.89	24
C3 Michigan	-16.54	(16.27)	--	0.31	83
C3 Texas	+ 0.22	(3.59)	--	0.95	249

Naive specification without state context (Appendix Table A5, model C1) yielded a sign reversal driven by cross-state confounding; see Discussion

## Discussion

This study yields 3 principal findings. First, political partisanship was associated with routine childhood vaccination coverage across an 11-year state panel after adjustment for educational attainment, income, employment, and insurance coverage. Second, this association appeared stable over time rather than progressively worsening, suggesting a long-standing structural pattern rather than a purely recent phenomenon. Third, when political alignment and educational attainment were considered jointly, the lowest mean measles-mumps-rubella coverage was observed in the low-education, high-Republican-vote-share stratum, identifying a potentially vulnerable policy-relevant subgroup with mean coverage below 88%. The highest mean coverage was observed in the low-education blue stratum rather than the high-education blue stratum, suggesting that in politically liberal states, factors beyond educational attainment—such as stronger school-entry mandate enforcement or higher community-level trust in public health agencies—may sustain coverage even where educational attainment is lower.

These findings complement the simulated risk framework advanced by Kiang and colleagues [1]. Their modeling analysis demonstrated the population-level consequences of sustained declines in routine childhood vaccination, including increased outbreak potential as coverage erodes. Our analysis extends that framework by showing that lower coverage was not randomly distributed across the United States but instead was patterned by political context. Together, these results suggest that outbreak vulnerability may be geographically concentrated in communities that are not only underimmunized but also situated within distinct political environments.

An important methodological consideration arises from recent county-level work by Dong and colleagues, who examined measles-mumps-rubella coverage trends across more than 2,000 US counties and reported that Republican-leaning counties had comparable or higher coverage rates in a 2017–2024 dataset [10]. This finding appears to contrast with our state-level result, in which higher Republican vote share was associated with lower coverage. Such scale-dependent divergence is consistent with the modifiable areal unit problem, in which associations estimated at different levels of geographic aggregation can differ in magnitude or direction [11]. State-level aggregation may mask within-state heterogeneity — for example, politically conservative counties within blue states may differ from those within red states in ways that alter or reverse the ecological association at the county level. Our 11-year panel extending to 2014 captures pre-2016 baseline political conditions that shorter panels do not, and our multi-vaccine comparison across 4 antigens provides breadth that single-vaccine county analyses cannot. Multi-scale surveillance — combining state-level panel models with state-context-controlled county analyses — is therefore necessary when characterizing political contextual influences on vaccination coverage.

Within the present revision, our county-level sequential adjustment ladder makes this scale-dependent divergence concrete. In the four-state 2023 cross-section, the political coefficient changes sign after the addition of percentage of non-Hispanic White population without simultaneous control for state context, reflecting the joint cross-state distribution of racial composition, political alignment, and vaccination coverage rather than a within-state reversal. When state context is restored through state fixed effects (model C2) or through state-stratified estimation (model C3), the coefficient returns to the negative direction observed at the state level. We therefore interpret a single county-level cross-sectional regression without state-aware modeling as inadequate for inference about the political-coverage relationship; multi-scale, state-aware analyses are required for valid contextual inference, and the sequential-adjustment ladder is presented in Appendix Table A5 to make this dependence explicit.

The current findings are also consistent with a growing literature linking vaccination to political context, although much of that literature has emphasized COVID-19 vaccination, legislative behavior, or policy polarization rather than routine childhood coverage across a long multiyear state panel [4–8, 12–18]. Prior work on exemptions and vaccine refusal has shown that school-entry policy environments and social attitudes are relevant to vaccination uptake and outbreak vulnerability [12–15]. The present study adds to that literature by suggesting that routine childhood vaccination, like more overtly politicized health behaviors, tracks broader political context even after measured socioeconomic factors are taken into account. That distinction is important because routine childhood vaccines are usually treated as standard pediatric preventive care rather than as an episodic partisan controversy.

Several mechanisms operating at the state level could plausibly contribute to this ecological pattern. Prior individual-level survey research has linked political identity to differential trust in public health institutions, varying perceptions of scientific credibility, and divergent responses to vaccine communication [7, 8]. At the aggregate level, differences in school-entry mandate strictness, media ecosystem composition, and local exemption climates may also contribute to the observed between-state coverage variation [17, 19, 20]. These pathways remain hypotheses that the present ecological design cannot directly test, but they illustrate plausible channels through which political context at the state level could co-occur with differential vaccination coverage. An exploratory interaction analysis found that the negative association between Republican vote share and MMR vaccination coverage was stronger in states with higher non-Hispanic White population share (interaction b=-0.33; *P*<.001; Appendix Table A6). This is consistent with the notion that political signaling on health behaviors operates differently across racial-demographic contexts and should not be reduced to a single political effect.

These findings have practical implications for immunization program design. Because the adjusted R^2^ of 0.31 reflects the combined contribution of political, educational, and economic factors, the unique variance attributable to political context alone is likely smaller, and political partisanship should be understood as one of several correlated contextual influences rather than a dominant driver. First, federal and state public health communication strategies may benefit from accounting for the local political environment rather than assuming that identical messages will perform similarly across jurisdictions. Second, vaccination surveillance could be stratified by political-contextual as well as socioeconomic characteristics to identify communities at elevated risk of undercoverage. Third, communities characterized by lower educational attainment and more conservative political alignment may represent priority targets for tailored outreach, provided that such strategies are designed to reduce trust barriers rather than reinforce partisan framing. More broadly, immunization policy may need to engage communication, trust, and institutional legitimacy alongside technical access measures. Health departments may also benefit from partnering with clinicians, schools, and locally trusted messengers who can translate vaccine recommendations in ways that fit community norms without amplifying partisan identity. Recent national trends in vaccination exemptions further reinforce the urgency of context-sensitive surveillance, as county-level exemption rates have risen in multiple jurisdictions since 2020 [21].

### Limitations

This study has several limitations. The ecological design means that state-level associations should not be attributed to individual parents or households without individual-level verification [11]. Harmonized state-level measles-mumps-rubella and hepatitis B coverage for 2022 were not available in the recoverable source set, constraining outcome-specific continuity for that year; no states had recoverable 2022 state-level measles-mumps-rubella data in the harmonized panel, indicating a systematic reporting gap rather than selective missingness. The county-level sensitivity analysis covered 4 states and should be interpreted as a robustness check rather than external validation. Political, economic, and educational indicators were correlated, so residual collinearity remains a concern despite the model’s stability. The primary model did not account for potential spatial autocorrelation among geographically proximate states, which could lead to underestimation of standard errors; however, the magnitude and precision of the primary coefficient suggest that moderate inflation of standard errors would be unlikely to reverse the overall finding. This study did not include state-level nonmedical exemption policy status as a covariate. Because exemption policy is itself shaped by the political environment, controlling for it could constitute overadjustment for a mediator on the pathway between political context and vaccination coverage; nevertheless, exemption policy changes during the study period, particularly the elimination of nonmedical exemptions in several states [16], may account for a portion of the observed association, and future analyses incorporating policy indicators as potential mediators would help disentangle these pathways. Unmeasured state characteristics including religious composition, media consumption, and provider density may account for additional variance. The county-level analyses were based on a 2023 cross-section across four states and therefore could not exploit within-county temporal variation. The naive cross-sectional specification (Appendix Table A5, model C1) produced a sign reversal driven by cross-state heterogeneity in the joint distribution of racial composition, political alignment, and vaccination coverage. State-context-controlled specifications (county fixed effects, state-stratified) recovered the expected direction. We therefore caution against interpreting any single county-level cross-sectional regression in isolation; multi-scale, state-aware analyses are required for valid contextual inference. Although racial composition is now included as a covariate in the primary specification, state-aggregated race covariates cannot capture within-state racial heterogeneity and ecological associations cannot identify individual-level behavior; the interaction and stratified analyses (Appendix Table A6) suggest that the political effect varies across the racial composition of states, in a way that should not be reduced to a single political coefficient. Finally, racial-composition values for 2024 were carried forward from 2023 because the ACS 1-year 2024 release was not yet available at the time of analysis; this affects only the 2024 panel year.

## Conclusions

State-level political context was associated with childhood vaccination coverage in a stable, long-term pattern across the United States from 2014 to 2024. Efforts to sustain routine immunization may benefit from accounting for the political context in which public health programs operate.

## Supplementary Information

Supplementary Material 1.

## Data Availability

All data analyzed in this study are publicly available. Vaccination coverage data were obtained from the Centers for Disease Control and Prevention. Socioeconomic data were obtained from the American Community Survey. Political data were obtained from public election records. The harmonized analytic panel and analysis code are available from the corresponding author on reasonable request.
