# Supplementary material for: Political polarization, educational attainment, and childhood vaccination coverage in the United States, 2014–2024: a cross-sectional study
Source: BMC Public Health. 2026 Jul 23;26:3006. doi: 10.1186/s12889-026-27675-9 (PMC13679233; doi:10.1186/s12889-026-27675-9)
Supplement: Supplementary file 1 [file 12889_2026_27675_MOESM1_ESM.doc]

**Technical Appendix**

*Supplementary Tables A1-A6 for "Political Affiliation, Race, and Routine Childhood Vaccination Coverage in the United States, 2014-2024"*

Wei Lin, Mayo Clinic. | BMC Public Health major revision | 2026-04-27

All numerical values in this appendix are reproduced from the analysis pipeline (scripts 01-04 in the reproducibility ZIP). The harmonized data files have been fingerprinted (SHA-256) and the seeds for any stochastic step are fixed. Regression outputs are unstandardized OLS coefficients (b), standard errors (SE), 95% confidence intervals (CI), and 2-sided P values. n is the analytic sample size after listwise deletion of covariates.

This appendix supplements the main text. The PRIMARY county-level sensitivity evidence is presented in Table 3 of the main text; Tables A1-A6 below provide the diagnostic and full-detail support for the manuscript's claims.

**Table A1. Variance Decomposition of Republican Vote Share**

Reviewer 2's third remark observed that Republican presidential vote share is highly stable within states, raising concern about the appropriateness of state fixed effects absorbing the exposure of interest. The unconditional intraclass correlation coefficient (ICC), treating state as the random-intercept grouping factor, quantifies the share of total variance attributable to between-state differences. ICC values approaching 1 indicate that within-state temporal variation is negligible relative to between-state differences.

| **Component** | **Value** | **Interpretation** |
| --- | --- | --- |
| Total observations | 508 state-year cells (51 states x 11 years - missing) |  |
| Between-state variance (sigma^2_between) | 0.014228 | state-level component |
| Within-state variance (sigma^2_within) | 0.000124 | year-to-year component |
| Between-state SD (sigma_between) | 0.1193 | approx 0.119 |
| Within-state SD (sigma_within) | 0.0111 | approx 0.011 |
| Variance ratio (between / within) | 115.17 | approx 115:1 |
| Intraclass correlation (ICC) | 0.9914 | approx 0.991 |

Implication: 99.1% of variance in Republican vote share is between states; only 0.9% is within-state year-to-year movement. State fixed effects therefore absorb essentially all of the exposure variance, leaving the within-state residual unable to identify the political-coverage relationship. The primary specification uses year fixed effects only; two-way (state and year) fixed effects are reported as a sensitivity analysis (Table A2), and a Mundlak decomposition explicitly separates between-state and within-state components (Table A3).

**Table A2. Two-Way Fixed Effects Sensitivity**

Two-way fixed-effects specifications are reported here for completeness. M3 adds state fixed effects to the primary year-FE model; M3b additionally permits each state its own linear time trend. As anticipated by the variance decomposition (Table A1), the political coefficient becomes statistically indistinguishable from zero with very wide CIs once state means are absorbed.

| **Specification** | **b (rep_share)** | **SE** | **95% CI** | **P** | **n** | **R^2** |
| --- | --- | --- | --- | --- | --- | --- |
| M1 (PRIMARY: year FE + race + log income) | -10.90 | (2.95) | -16.69, -5.10 | <.001 | 406 | 0.312 |
| M3 (two-way FE: state + year) | -0.90 | (14.25) | -28.93, +27.13 | 0.95 | 406 | 0.568 |
| M3b (state FE + state-specific linear trends) | -1.98 | (19.64) | -40.62, +36.66 | 0.92 | 406 | 0.611 |

Reading: M3 b = -0.90 (95% CI, -28.93 to +27.13; P = .95) and M3b b = -1.98 (95% CI, -40.62 to +36.66; P = .92) are wide-CI null results consistent with the within-state exposure being too compressed (within-state SD = 0.011) to identify the political effect. These specifications confirm rather than contradict the primary finding: within-state year-to-year shocks in Republican vote share carry essentially no signal for childhood vaccination coverage. The two-way FE model is reported here as a Reviewer 2 due-diligence check, not as a competing primary specification.

**Table A3. Mundlak Decomposition of Republican Vote Share**

The Mundlak (1978) decomposition replaces the original time-varying exposure with two orthogonal components: the state-mean projection (rep_state_mean, the persistent between-state component) and the within-state deviation (rep_within = rep_share - rep_state_mean). The two components are entered jointly. The primary covariate set is retained.

| **Component** | **b** | **SE** | **95% CI** | **P** | **n** | **R^2** |
| --- | --- | --- | --- | --- | --- | --- |
| rep_state_mean (between-state component) | -11.16 | (3.02) | -17.10, -5.21 | <.001 | 406 | 0.312 |
| rep_within (within-state deviation) | -4.60 | (16.48) | -37.01, +27.81 | 0.78 | 406 | 0.312 |

Reading: the between-state component carries the entire signal (b = -11.16; 95% CI, -17.10 to -5.21; P < .001), confirming that the political-coverage association is a between-state phenomenon. The within-state deviation is a wide-CI null (b = -4.60; 95% CI, -37.01 to +27.81; P = .78), consistent with Table A1's variance decomposition. We interpret the political-coverage relationship as a between-state association, which is the substantive question of interest for state-aggregated public-health policy.

**Table A4. Persistence-Classification Sensitivity (Definition A vs Definition B)**

Persistence classification restricts the panel to states with consistent presidential two-party vote outcomes. Definition A spans four elections (2012, 2016, 2020, 2024) and was selected because the state vaccination panel begins in 2014, immediately following the 2012 election; Definition A yields 21 always-Republican, 20 always-Democratic, and 10 swing states. Definition B spans the modern hyperpolarization era of three elections (2016, 2020, 2024) and yields 24 always-Republican, 20 always-Democratic, and 7 swing states. Both windows were pre-specified to avoid post-hoc selection.

| **Quantity** | **Definition A (4 elections, 2012-2024)** | **Definition B (3 elections, 2016-2024)** |
| --- | --- | --- |
| Composition | 21 always-R / 20 always-D / 10 swing (2012-2024 anchor) | 24 always-R / 20 always-D / 7 swing (2016-2024 anchor) |
| Subset N (state-year) | n = 326 (always-R + always-D) | n = 350 (always-R + always-D) |
| Welch t (unadj. MMR, R vs D) | t = -6.82; P = 3.4e-11 | t = -6.93; P = 1.6e-11 |
| Adjusted b (rep_share) | -14.30 | -13.17 |
| SE | (3.35) | (3.24) |
| 95% CI | -20.90, -7.70 | -19.54, -6.79 |
| P value | 2.7e-05 | 6.0e-05 |
| R^2 | 0.341 | 0.324 |

Reading: both persistence-restricted estimates are stronger than the all-state primary estimate of -10.90, and the Welch t tests of unadjusted coverage are highly significant (P < 1e-10) under both windows. The convergent direction across two pre-specified windows reduces concern about post-hoc selection and is consistent with the hypothesis that sustained political alignment is the more interpretable contextual exposure. The use of two windows (rather than one) is a robustness device: stability across anchors strengthens external validity.

**Table A5. County-Level Sequential Adjustment Ladder + State-Context-Controlled Models**

Reviewer 2's central concern was the apparent sign reversal of the political coefficient in a naive county-level cross-section. We address this with a sequential adjustment ladder (C1a-C1e) that documents each covariate's incremental contribution, alongside the state-context-controlled specifications (C2 with state fixed effects; C3 stratified by state) that constitute the PRIMARY county-level evidence.

**A5.1 Sequential Adjustment Ladder (4-state 2023 cross-section, n = 410)**

| **Step** | **Specification** | **b** | **SE** | **95% CI** | **P** | **n** | **R^2** |
| --- | --- | --- | --- | --- | --- | --- | --- |
| C1a | year FE + rep_share only | -0.28 | (2.54) | -5.27, +4.72 | 0.91 | 410 | 0.000 |
| C1b | + log_income | +2.94 | (2.66) | -2.30, +8.17 | 0.27 | 410 | 0.030 |
| C1c | + bachelor's, employment, uninsured (original SES) | -6.95 | (2.66) | -12.17, -1.72 | 0.009 | 410 | 0.248 |
| C1d | + pct_nhw | +20.72 | (3.22) | +14.40, +27.05 | <.001 | 410 | 0.450 |
| C1e | + pct_nhb + pct_hispanic (full race) | +21.38 | (3.22) | +15.06, +27.71 | <.001 | 410 | 0.468 |

Reading: the political coefficient is essentially zero with year FE alone (C1a, b = -0.28; P = .91); marginally positive after adding log income (C1b, b = +2.94; P = .27); negative after adding the conventional SES set (C1c, b = -6.95; P = .009); strongly positive after adding % non-Hispanic White (C1d, b = +20.72; P < 1e-9); and remains positive with the full race set (C1e, b = +21.38; P < 1e-10). The C1c-to-C1d sign reversal is therefore a property of the joint cross-state distribution of racial composition, political alignment, and vaccination coverage, not a within-state reversal. We interpret it as an instance of the modifiable areal unit problem (MAUP) described by Dong et al. (JAMA 2025): scale-dependent inference about contextual exposures is an established methodological hazard that requires state-aware modeling.

**A5.2 State-Context-Controlled Specifications (PRIMARY county-level evidence)**

When state context is restored - either through state fixed effects (C2) or through state-stratified estimation (C3) - the coefficient returns to the negative direction observed at the state level. These are the specifications presented in Table 3 of the main text and constitute the substantive county-level finding.

| **Specification** | **b** | **SE** | **95% CI** | **P** | **n** |
| --- | --- | --- | --- | --- | --- |
| C2: county OLS + state fixed effects (4-state cross-section) | -4.32 | (3.31) | -10.84, +2.19 | 0.19 | 410 |
| C3 California (within-state cross-section) | -9.89 | (8.11) | -26.20, +6.41 | 0.23 | 54 |
| C3 Maryland (within-state cross-section) | -1.02 | (7.46) | -16.75, +14.71 | 0.89 | 24 |
| C3 Michigan (within-state cross-section) | -16.54 | (16.27) | -48.94, +15.86 | 0.31 | 83 |
| C3 Texas (within-state cross-section) | +0.22 | (3.59) | -6.85, +7.29 | 0.95 | 249 |

Reading: three of four within-state estimates are negative; the Texas estimate is +0.22 with P = .95, which we interpret as an effective null rather than as evidence of an opposite within-state association. The Texas county set is dominated by border counties with uniformly high MMR vaccination coverage near 97%, and within-Texas variation in coverage is too compressed to carry signal (R^2 = 0.123). We therefore report no evidence of an opposite within-state association in any of the four sampled states. The state-FE specification (C2) yields b = -4.32 (95% CI, -10.84 to +2.19; P = .19), directionally consistent with the state-level finding although the four-state, single-year cross-section provides limited within-state degrees of freedom.

**Table A6. Race-by-Politics Interaction (M7) and NHW Tertile Stratification (M8)**

Reviewer 1 raised the question of whether the political-coverage association varies across the racial composition of states. We address this with a continuous-by-continuous interaction model (M7: rep_share x % non-Hispanic White) and a complementary stratified model (M8: separate political coefficients within tertiles of state-level % non-Hispanic White). All M7/M8 models include the primary covariate set (year FE, log income, bachelor's, employment, uninsured).

**A6.1 M7 -- Continuous Interaction Model (n = 406)**

| **Term** | **b** | **SE** | **95% CI** | **P** |
| --- | --- | --- | --- | --- |
| rep_share (main effect) | +13.88 | (6.32) | +1.45, +26.31 | 0.03 |
| % non-Hispanic White (main effect) | +0.16 | (0.03) | +0.09, +0.23 | <.001 |
| rep_share x % non-Hispanic White (interaction) | -0.33 | (0.08) | -0.48, -0.18 | <.001 |

Reading: the interaction coefficient is negative (b = -0.33; 95% CI, -0.48 to -0.18; P = 1.4e-5), indicating that the political-coverage association is more strongly negative in states with higher non-Hispanic White population share. R^2 = 0.345 for the M7 specification.

**A6.2 M8 -- NHW Tertile Stratified Models**

States were assigned to tertiles of state-mean % non-Hispanic White (low, middle, high). The primary specification was re-fitted within each tertile.

| **Stratum** | **b** | **SE** | **95% CI** | **P** | **n** |
| --- | --- | --- | --- | --- | --- |
| Low NHW tertile | -13.81 | (5.11) | -23.92, -3.70 | 0.008 | n = 136 |
| Middle NHW tertile | -6.89 | (5.78) | -18.32, +4.55 | 0.24 | n = 134 |
| High NHW tertile | -7.61 | (5.33) | -18.17, +2.95 | 0.16 | n = 136 |

Reading: all three tertile-specific estimates are negative (low: b = -13.81, P = .008; middle: b = -6.89, P = .24; high: b = -7.61, P = .16). The loss of nominal statistical significance in the middle and high tertiles reflects halved sample size (n approx 135) rather than directional disagreement; pooled across tertiles the direction is consistent with the M7 interaction result. We interpret the M7/M8 results jointly as evidence that the political-coverage association does not reduce to a single political coefficient and varies across racial-demographic contexts, which we discuss in the main text.

**Diagnostic Notes (D1-D5)**

A pre-flight diagnostic suite (scripts code/diag_c1_sign_flip.py and results/diagnostic_C1_signflip.md) audited five potential causes of the C1 sign reversal before any reviewer-facing analysis was finalized.

| **Diagnostic** | **Status** | **Finding** |
| --- | --- | --- |
| D1 Unit-of-measure audit | PASS | State and county rep_share both on [0,1] proportion scale; consistent. |
| D2 Race-variable provenance | PASS | pct_nhw varies across counties within each state (within-state SD: CA 19.5, MD 20.7, MI 9.0, TX 20.6); not state-broadcast. |
| D3 Panel-structure audit | PASS | 411 county-year rows from 2023 single-year cross-section; C0 (n=411) and C1 (n=410) on the same row set (one row dropped by listwise deletion). |
| D4 Collinearity diagnostics | PASS | VIF max (excluding constant) = 2.93 (pct_uninsured); corr(rep_share, pct_nhw) = 0.328 in county sample. No multicollinearity flag. |
| D5 Sequential adjustment trace | RESOLVED | Sign change driven specifically by addition of pct_nhw (C1c -> C1d); coefficient returns to negative direction with state fixed effects (C2) and state-stratified estimation (C3). Documented as MAUP. |

All five diagnostics returned interpretable results before the C2/C3 framing was finalized. The diagnostic report is reproduced verbatim in the reproducibility ZIP (results/diagnostic_C1_signflip.md).
